# Interfacial magnetic-phase transition mediated large perpendicular magnetic anisotropy in FeRh/MgO by a heavy transition-metal capping

**DOI:** 10.1038/s41598-018-24977-w

**Published:** 2018-05-02

**Authors:** Dorj Odkhuu

**Affiliations:** Department of Physics, Incheon National University, Incheon, 22012 South Korea

## Abstract

Stacking a magnetic memory junction in spintronic devices necessarily involves making contacts with a transitional-metal capping electrode. Herein, by means of first-principles calculations, we reveal the importance of heavy transition-metal capping on magnetic-phase transition from antiferromagnetic (AFM) to ferromagnetic (FM) order and the large perpendicular magnetic anisotropy (PMA) found in Ta-capped FeRh films on MgO substrate. While magnetization of FeRh films reorients from in-plane to PMA when in contact with MgO, the presence of Ta capping further enhances the magnitude of the PMA energy by at least five times. This large PMA is associated with the AFM-FM transition at the interface, which in turn modifies the out-of-plane Fe 3*d* orbital states through the hybridization with the strong spin-orbit coupled Ta 5*d* orbitals. Furthermore, the magnetic-phase transition at the interface is the result of the mutual mechanisms of the capping-induced volume/tetragonal expansion in the interfacial FeRh layers and the competition between the direct and indirect exchange interactions. These findings suggest that Ta/FeRh/MgO multilayers may represent highly favourable memory materials with net interfacial ferromagnetism and large PMA in antiferromagnet spintronics.

## Introduction

Explorations of magnetic tunnel junctions (MTJs) typically composed of the heavy transition-metal (HTM) capped ferromagnets (FM) on insulating MgO (HTM/FM/MgO) have been very successful for their applications in spin-transfer torque (STT) memory, owing to their large magnetoresistance (MR) and perpendicular magnetic anisotropy (PMA)^1^. Nevertheless, intense research efforts continue to seek reductions in the critical current density, ${I}_{c}$, which are required for magnetization switching of a free FM layer in MTJs, while retaining the thermal stability, Δ. Δ is maintained by the large PMA according to ${\rm{\Delta }}=KV/{k}_{B}T$, where *K*, *V*, ${k}_{B}$, and *T* are the anisotropy, volume, Boltzmann constant, and temperature, respectively^2^. On the other hand, low saturation magnetization ${M}_{s}$ favours the reduction of ${I}_{c}$ according to ${I}_{c}=\frac{2e}{\hslash }\frac{\alpha }{\eta }{M}_{s}V({H}_{k}+2\pi {M}_{s})$, where $\alpha$, $\eta$, and ${H}_{k}$ represent the Gilbert damping coefficient, spin polarization factor, and Stoner-Wolfarth switching field, respectively^3^. Most materials proposed for memory applications are soft magnets, i.e., ${H}_{k}\ll {M}_{s}$, and thus ${I}_{c} \sim {M}_{s}^{2}V$. The utilization of low or zero net magnetization materials, i.e., antiferromagnetic (AFM) materials, preferably with large PMA, in MTJs could thus provide an alternative way to minimize ${I}_{c}$ and maximize Δ at the same time^4,5^ as well as reduce stray fields in real devices^6,7^.

In the context of AFM-based MTJs, B2-ordered FeRh alloys have been recently regarded as potential candidates because they can be epitaxially grown on MgO^6–8^ and BaTiO^3,9,10^. Furthermore, a giant room-temperature MR effect of a resistivity change of Δ*R*/*R* ~50% has been discovered in these alloys, accompanying an unusual first-order transition from AFM to FM phases via an applied magnetic field^11^. A similar magnetic-phase transition can also be driven by heating to just above room temperature (~350 K), which is correlated with a volume expansion of ~1% and a Rh-site net moment of ~1 *μ*_*B*_ without a change in the crystallographic structure^12–14^. In more recent experiments, thin films of FeRh have been identified to even exhibit rich emergent phenomena such as thermal and electric-field control of magnetic-phase transitions^9,10^ and room-temperature bistable AFM formation^6,7^. Remarkably, ^57^Fe conversion electron Mossbauer spectroscopy experiments^8^ and first-principles calculations^15^ reveal the strain and electric-field reversal induced spin reorientation of the easy magnetization axis at the magnetic-phase transition of FeRh films on MgO and BaTiO_3_ substrate. These results suggest that FeRh thin films have potential not only for STT technologies but also for utilization in novel emerging memory applications such as heat-assisted magnetic recording (HAMR)^16^ and magnetoelectric random access memory (MeRAM)^9,10^.

Such an intriguing feature of FeRh can be synergistically controlled by surface-side doping or capping with TM elements^16,17^. The magnetic-phase transition temperature (${T}_{t}$), for instance, is strongly altered over a wide temperature range 100 ≤ *T*_*t*_ ≤ 600 K with TM substitutions (ref.^17^ and references therein). Through X-ray magnetic circular dichroism and photoemission electron microscopy experiments, the coexistence of the two phases, with stable interfacial FM domains at room temperature, has also been observed in TM-capped FeRh/MgO films^18,19^. Although there have been numerous subsequent studies for TM-capped FeRh films, the underlying physical origin for the interfacial ferromagnetism and the early stage magnetic-phase transition remains unclear. In addition, these results further urge a vital exploration of the effects of HTM capping on the magnetic anisotropy of FeRh films, which remain unexplored but essential in actualizations of AFM materials in memory technologies.

Herein, the present study using electronic structure first-principles calculations aims to provide physical insights into the interface ferromagnetism and large PMA found in Ta-capped FeRh films on MgO. Although the presence of MgO substrate reorients the in-plane magnetization of FeRh films to PMA, a substantial enhancement in the magnitude of the magnetic anisotropy energy (MAE) is demonstrated by capping with Ta layers. Single-particle energy spectra analyses with spin-orbit Hamiltonian matrix elements reveal that the underlying mechanism is the interplay between the out-of-plane *d*-orbital states, ${d}_{{z}^{2}}$ and ${d}_{xz,yz}$, of the Ta 5*d*–Fe 3*d* hybridized orbitals with large spin-orbit coupling. We further propose that the AFM–FM transition at the interface is driven by the mutual mechanisms of the capping-induced volume/tetragonal expansion in the interfacial FeRh layers and the competition between the direct and indirect exchange interactions within the interfacial Fe plane mediated by the hybridization with the spin-polarized Ta 5*d* orbitals.

## Results and Discussion

**Figure 1:**
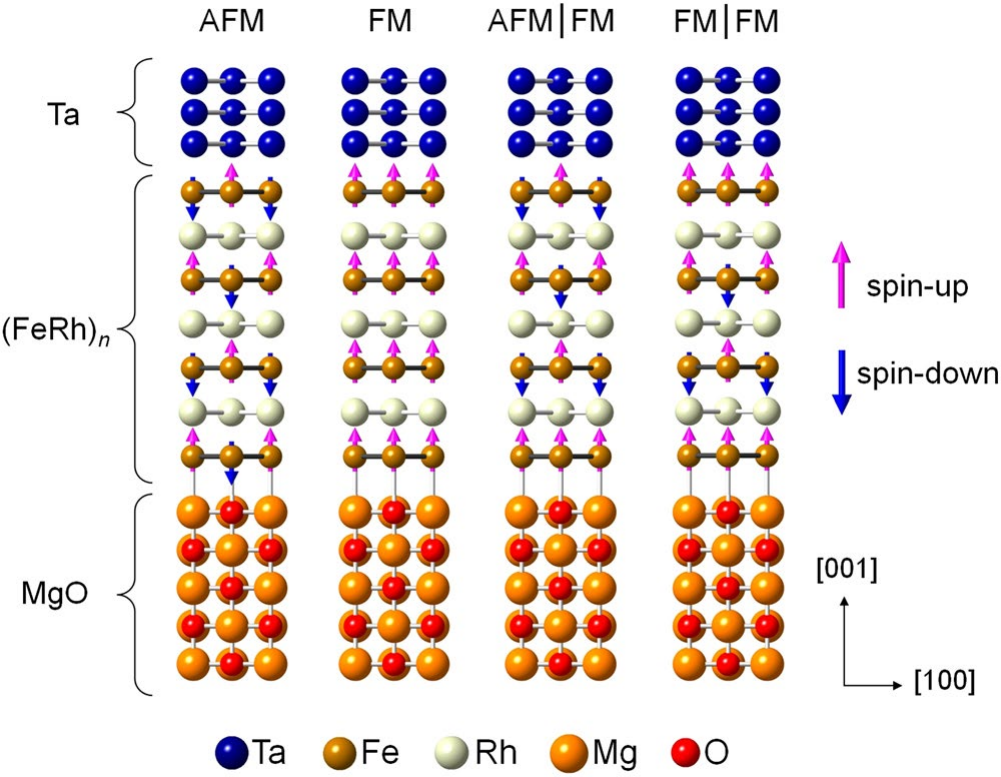
Representation of the atomic structure of Ta/FeRh/MgO multilayers for the different magnetic configurations: AFM-II, FM, AFM(I_Ta_)/FM(I_O_), and FM(I_Ta_)/FM(I_O_). The larger blue, brown, grey, orange, and smaller red spheres are Ta, Fe, Rh, Mg, and O atoms, respectively. The red upward and blue downward arrows indicate the spin orientations of Fe atoms. The letter *n* in the subscript denotes the number of FeRh unit cell layers.

It is known that in most HTM/FM/MgO multilayers, the film thicknesses of HTM and FM layers, especially FM layers, play an important role in determining the MAE. In experiments, for example, the typical thickness of FM layers that exhibit PMA in Ta/CoFeB/MgO is within the range of 0.5–1.2 nm^1^. In accordance with a realistic situation, we explore supercells composed of 2–7 unit cell (*uc*) FeRh layers (*n*), which are approximately 0.6–3 nm thick, on five atomic layers (ALs) of the MgO substrate capped by three ALs of Ta atoms. We adopted the Ta layers as a capping electrode based on practical usage in most experimental works undertaken thus far, where the presence of the Ta layers is crucial in determining the PMA^1,20^. For instance, PMA has been achieved exclusively in Ta/CoFeB/MgO and not in Ru/CoFeB/MgO multilayers^20^. Considering the lack of generality and reality, the results corresponding to the thicknesses of 1 *uc* FeRh and 1–2 Ta ALs are excluded. For each *n*, four distinct magnetic structures were taken into account to identify the most stable phase: entirely Type-II AFM (AFM-II) and FM and reconstructed AFM-II with an FM layer at the interface next to MgO, denoted AFM(I_Ta_)/FM(I_O_), and next to both the MgO and Ta layers, denoted FM(I_Ta_)/FM(I_O_); see Fig. 1. The other spin-antiparallel configurations, i.e., Type-I (or A-AFM) and Type-III (or C-AFM), have been excluded because they were found to have relatively high energies than those for the AFM-II and FM phases^21^. The preferred adsorption sites of Fe atoms were atop O at the FeRh/MgO interface and a hollow site at the Ta/FeRh interface, which are analogues to a Ta/Fe/MgO multilayer^22^. An experimental lattice constant (4.212 Å) for MgO was adopted for the in-plane lattice of supercells, which was matched to the optimized bulk lattices of AFM-II and FM FeRh within 0.5 and 1.1%, respectively^5^. As reference systems, we have also taken into account the FeRh and FeRh/MgO films without Ta capping, for which FM(I_V_) refers to the FM interface next to a vacuum region (V).

**Figure 2:**
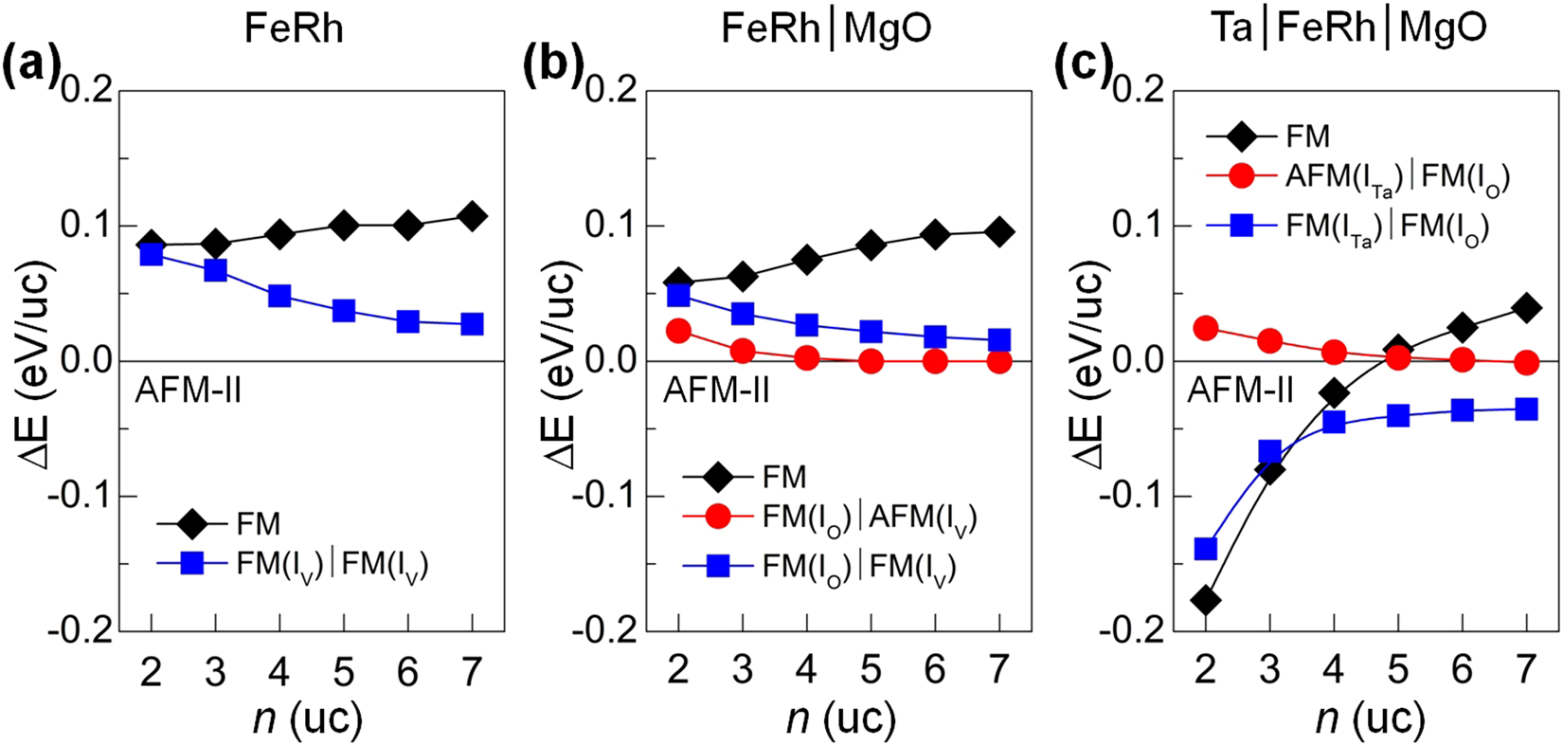
Relative energy ΔE versus the numbers of FeRh unit cell layers (*n*) for the different magnetic structures shown in Fig. 1 with respect to the AFM-II phase for (**a**) FeRh, (**b**) FeRh/MgO, and (**c**) Ta/FeRh/MgO multilayers.

The relative energies Δ*E* of the aforementioned magnetic configurations with respect to the AFM-II phase are shown in Fig. 2(a–c) for FeRh, FeRh/MgO, and Ta/FeRh/MgO multilayers, respectively. For FeRh films, AFM-II is the most stable phase and serves as a ground state phase in low temperature experiments^6–8^. In the AFM-II phase, the Fe atoms within the *xy*-plane prefer spin-antiparallel coupling to the nearest neighbours through the Zener-type direct superexchange interaction and spin-parallel coupling to next-to-nearest neighbours through a nonmagnetic mediation (Fe-Rh-Fe) by the Goodenough-Kanamori-Anderson (GKA) mechanism^21^. In the GKA rule, the Fe atoms in the [111] and [110] directions prefer the spin-antiparallel and spin-parallel couplings, respectively, owing to the Fe-Rh-Fe angles of 180° and near 90° (a more detailed explanation is provided in ref.^21^). When in contact with MgO, the AFM(I_V_)/FM(I_O_) phase is favoured, as its Δ*E* decreases with *n* and reaches that for the AFM-II phase at *n* ≥ 5 *uc*. The existence of such FM layers at the FeRh/MgO interface has been reported in recent experiments^23^ and discussed in terms of the broken GKA rule and Fe 3*d*–O 2*p* hybridization by theoretical studies^5,21^.

On the other hand, distinctly different features appear in Ta/FeRh/MgO: either the AFM-II and AFM(I_Ta_)/FM(I_O_) are not favoured regardless of *n*, but the magnetic-phase transition from the entire FM to FM(I_Ta_)/FM(I_O_) phase occurs at *n* = 4 *uc*. As a generic rule, the saturation behaviour of Δ*E* is evident as the number of FeRh layers increases beyond *n* = 4 or 5 *uc*. Thus, for the results and discussion here and hereafter, we refer mainly to those corresponding to *n* = 5 *uc*, unless specifically mentioned. The FM ground state of the Fe(I_Ta_) layer can be attributed to the following mechanisms. First, stronger hybridization between the Ta 5*d* and Fe 3*d* orbital states (rather than Rh 4*d*–Fe 3*d*) occurs when the Ta capping is present owing to the feature of their common peak electronic structures (as discussed below). Second, the aforementioned GKA-type superexchange coupling is not applicable to the Ta/FeRh interface because of the missing Fe layer above the interfacial Ta layer, denoted as Ta(I). Third, a sufficiently large magnetism (~−0.3 *μ*_*B*_) of Ta(I) in antiparallel to that of the interfacial Fe layer would enable such a stable FM spin orientation within the underlying Fe(I_Ta_) atoms through an indirect exchange interaction mediation.

To a certain extent, the AFM → FM transition could correlate to a volume change of the interfacial FeRh *uc* layers in the presence of the Ta capping. The optimized tetragonal distortion *c*/*a* and interlayer distances (*d*_Fe-X_) at the Ta/FeRh/MgO interfaces are shown in Table 1, in comparison with those for FeRh and FeRh/MgO films. The interlayer distance between the interfacial Fe next to MgO, denoted as Fe(I_O_), and the top O plane in MgO, *d*_Fe-O_, is ~2.18 Å, which is comparable with that (2.145 Å) in bulk FeO but smaller than that (2.35 Å) in the Fe*/*MgO bilayer. In this interface (next to MgO), the *c*/*a* and *d*_Fe-Rh_ are 1.02 and 1.43 Å, respectively, which are slightly larger than the corresponding values (0.99 and 1.39 Å) of free-standing FeRh films. On the other hand, these parameters even further increase substantially at the other interface next to the Ta layers (*c*/*a* = 1.03 and *d*_Fe-Rh_ = 1.51 Å), leading to a significant volume expansion of the interfacial FeRh unit cell in the presence of Ta by ~4%. Note that the thermally driven magnetic-phase transition of FeRh accompanies a similar phenomenon of the volume expansion (~1%) without a change in the crystallographic structure, as mentioned previously^12–14^. Our calculations in bulk FeRh show that the volume increases from the AFM to the FM phase by ~1.2%, where the *c*/*a* values are 1.00 and 1.01 for the AFM and FM phase, respectively.

**Table 1:** Values of the *c*/*a* ratio and the interlayer distances (*d* in Å) at the interfaces for FeRh in the most stable AFM, FeRh/MgO in the AFM(I_V_)/FM(I_O_), and Ta/FeRh/MgO in the FM(I_Ta_)/FM(I_O_) phase. The letters I_O_ and I_Ta_ denote the interfaces next to the MgO and Ta layers, respectively. The same in-plane lattice (*a*) of 2.978 Å was employed for each system.

	*a*	*c*/*a*(I_O_)	*c*/*a*(I_Ta_)	*d*_Fe-O_(I_O_)	*d*_Fe-Rh_(I_O_)	*d*_Fe-Rh_(I_Ta_)	*d*_Fe-Ta_(I_Ta_)
FeRh	2.978	0.99	0.99	—	1.39	1.39	—
FeRh/MgO	2.978	1.02	0.99	2.18	1.43	1.39	—
Ta/FeRh/MgO	2.978	1.02	1.03	2.18	1.43	1.51	1.45

We next show the spin magnetic moments of the interface atoms along with the number of electrons in the majority-spin and minority-spin channels in Table 2. It is clear that the quantization of the charge density is more significant at the Ta/FeRh interface than at the FeRh/MgO interface. While the Fe atom interfacing with Ta, denoted as Fe(I_Ta_), loses (gains) its majority (minority) spin electron of 0.16*e* (0.56*e*), the Rh(I_Ta_) atom accumulates (depletes) 0.22*e* (0.23*e*) in the majority (minority) spin state. The extra minority-spin charge accumulation of Fe(I_Ta_) is mainly from the interface Ta, as the Ta atoms have less electronegativity (1.5) than the Fe atoms (1.83). As a result, the interface Ta (Rh) atoms have an induced moment of −0.32 (0.51) *μ*_*B*_ antiparallel (parallel) to the Fe(I_Ta_) moments. These induced moments are mainly confined to the interface layers but are quenched at the layers away from the interface. It is also noteworthy that such a sufficiently large magnetism of Rh atoms supports the magnetic-phase transition at the interface, as in FM bulk and films^24,25^.

**Table 2:** The numbers of electrons in the majority-spin $({n}_{\uparrow })$ and minority-spin $({n}_{\downarrow })$ states and spin magnetic moments (*µ*_*s*_) of the interface (I) atoms for FeRh/MgO in the most stable AFM(I_V_)/FM(I_O_) phase and Ta/FeRh/MgO in the most stable FM(I_Ta_)/FM(I_O_) phase. The letters I_O_ and I_Ta_ denote the interfaces next to the MgO and Ta layers, respectively. The letter I_V_ denotes the interface of FeRh/MgO next to the vacuum level, where the local spin states and magnetic moments of the Fe(I_V_) atom are shown.

	O(I)	Fe(I_O_)	Rh(I_O_)	Rh(I_Ta_)	Fe(I_V_/I_Ta_)	Ta(I)
**FeRh/MgO**						
${n}_{\uparrow }$	2.56	5.01	4.31	3.99	5.00	—
${n}_{\downarrow }$	2.51	1.94	3.63	3.96	1.84	—
*µ*_*s*_	0.04	3.04	0.67	0.02	3.16	—
**Ta/FeRh/MgO**						
${n}_{\uparrow }$	2.56	5.01	4.30	4.21	4.84	2.27
${n}_{\downarrow }$	2.51	1.94	3.64	3.73	2.40	2.59
*µ*_*s*_	0.04	3.04	0.66	0.51	2.44	−0.32

**Figure 3:**
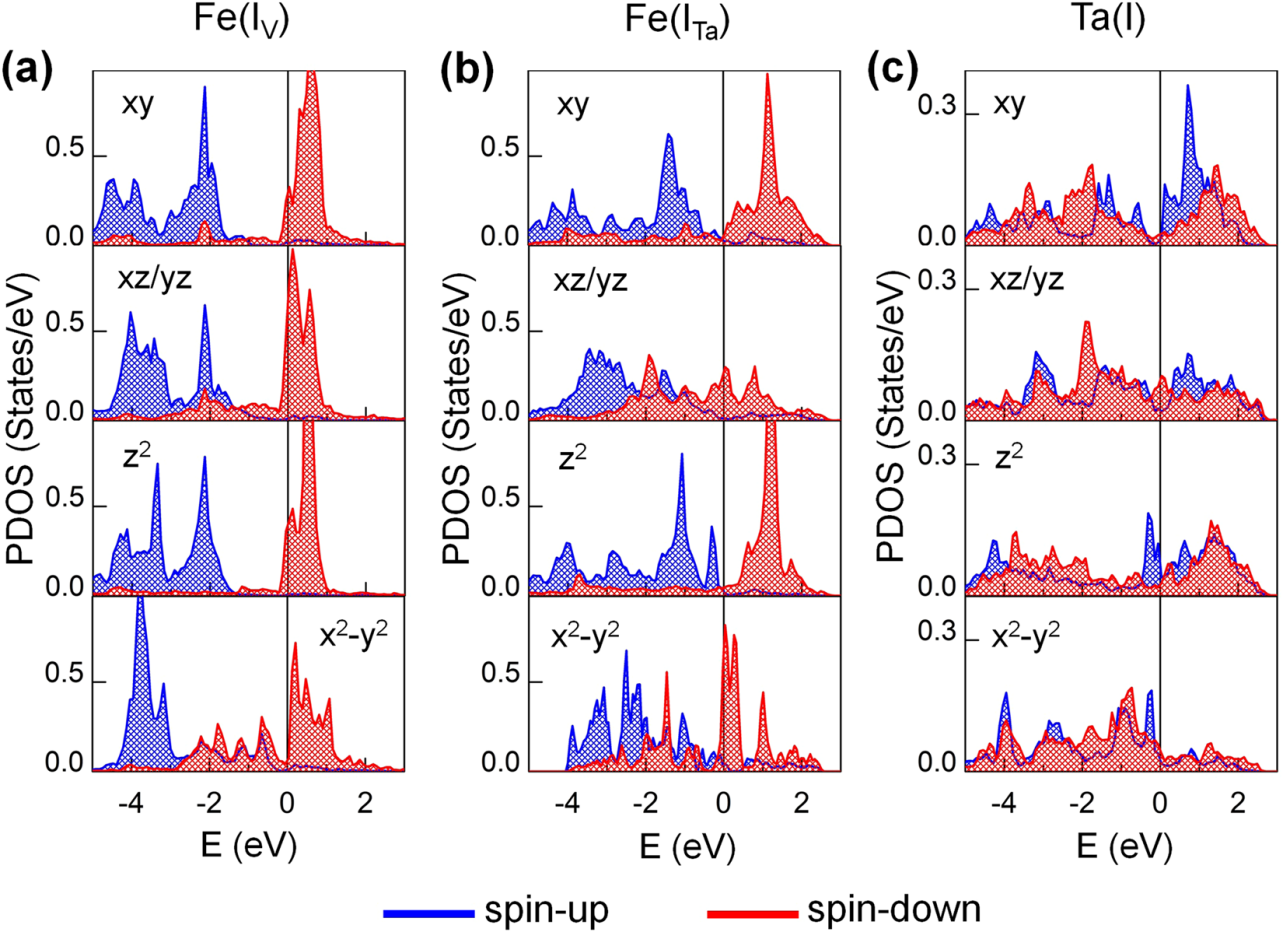
Spin and *d*-orbital decomposed PDOS of (**a**) Fe(I_V_) in FeRh/MgO and (**b**) Fe(I_Ta_) and (**c**) Ta(I) in Ta/FeRh/MgO multilayers. The blue and red areas represent the majority-spin and minority-spin states, respectively. The Fermi energy is set to zero energy.

To better understand the instability of ferromagnetism, we show the *d*-orbital projected density of states (PDOS) of the Fe(I_V_) atom in FeRh/MgO and Fe(I_Ta_) and Ta(I) atoms in Ta/FeRh/MgO in Fig. 3(a–c). The fully occupied majority-spin states of Fe(I_V_) shift towards the Fermi level in the presence of Ta capping, while the minority-spin states are greatly broadened and become more occupied. As a reflection of this reduced spin exchange splitting, the magnetic moment decreases significantly from the Fe(I_V_) to the Fe(I_Ta_) sites (Table 2). Notably, the coincidences of the minority-spin Fe(I_Ta_)- and Ta(I)-$\,{d}_{xz,yz}$ peak states around the Fermi level and the majority-spin Fe(I_Ta_)- and Ta(I)-$\,{d}_{{z}^{2}}$ peak states just below the Fermi level are apparent. This implies that the AFM-FM transition at the interface accompanies the strong out-of-plane orbital (${d}_{xz,yz}$ and ${d}_{{z}^{2}}$) hybridization between the Ta 5*d* and Fe 3*d* states. A similar argument applies to the FeRh/MgO interface, as addressed in previous *ab initio* calculations^5,22^.

**Figure 4:**
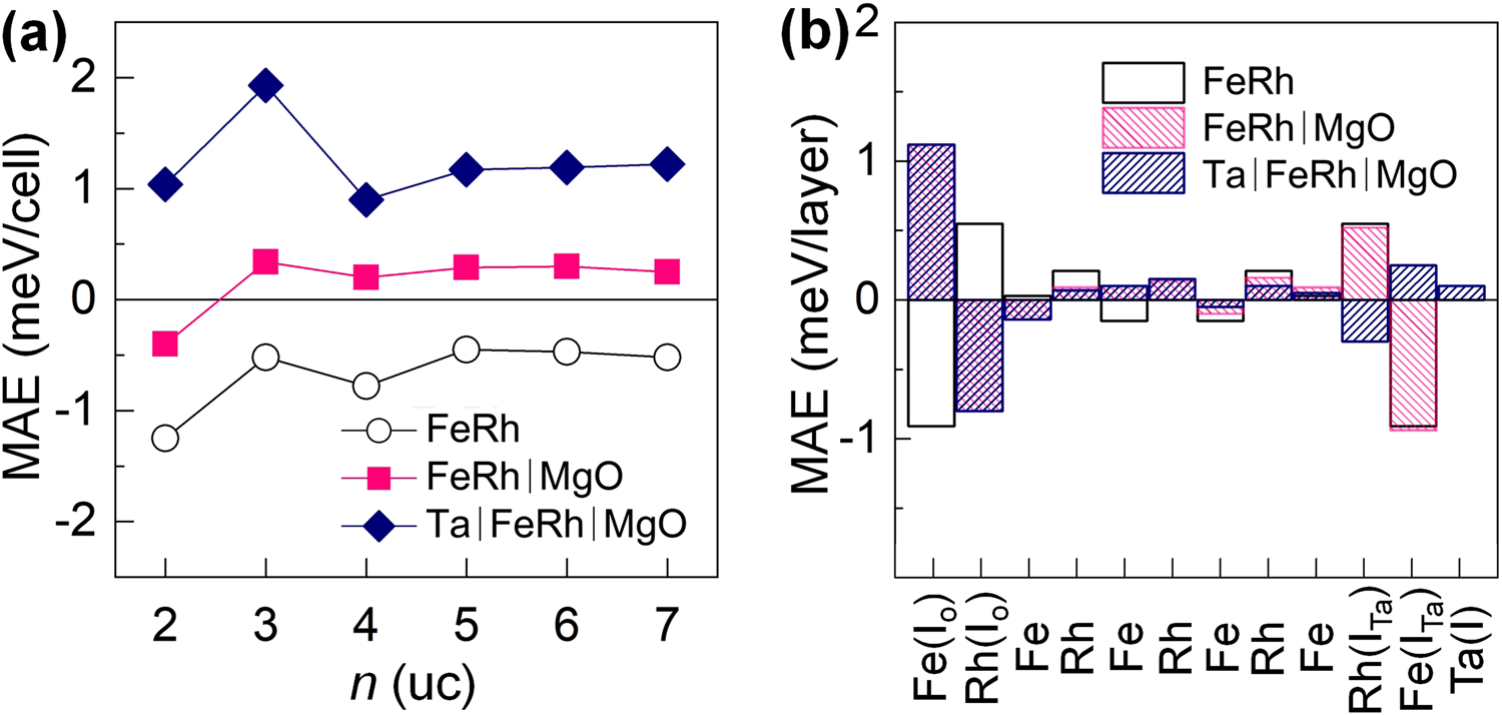
(**a**) Magnetic anisotropy energy (MAE) versus the number of FeRh unit cell layers (*n*) for FeRh (open black circles), FeRh/MgO (filled red squares), and Ta/FeRh/MgO (filled blue rhombi) multilayers. (**b**) Layer-resolved MAE for FeRh layers for FeRh (open black bars), FeRh/MgO (filled red bars), and Ta/FeRh/MgO (filled blue bars) multilayers with *n* = 5 *uc*. In (**b**), the letters I_O_ and I_Ta_ denote the interfaces next to the MgO and Ta layers, respectively.

Figure 4(a) shows the calculated MAE as a function of *n* for the Ta/FeRh/MgO multilayer for the most stable FM(I_Ta_)/FM(I_O_) phase (filled rhombi). The results for FeRh with the AFM-II phase (open circles) and FeRh/MgO with the AFM(I_V_)/AFM(I_O_) phase at *n* = 2 - 4 *uc* and AFM(I_V_)/FM(I_O_) phase at *n* ≥ 5 *uc* (filled squares) are also compared. The pristine FeRh films exhibit an in-plane magnetization, where MAE = −0.36 meV/cell, in agreement with previous *ab initio* calculations^20^. In the presence of MgO, this in-plane magnetization of FeRh films undergoes a transition into perpendicular magnetization, i.e., PMA. The underlying mechanism for this magnetization reorientation is associated with the AFM-FM transition at the interface, in addition to the orbital hybridization between the Fe ${d}_{{z}^{2}}$ and O *p*_*z*_ states^5,26^. We find that the MAE values of the *bulk* FeRh are −0.004 and 0.021 meV/Fe (−0.05 and 0.25 meV/12-Fe-atom cell or 5 *uc*) for the AFM and FM phases, respectively. Previous *ab initio* calculations indeed showed that the MAE of the interfacial Fe layer of FeRh/MgO bilayer is a positive in the FM phase but a negative in the AFM phase^5^. More remarkably, as shown in Fig. 4(a), the presence of the Ta capping further enhances the PMA of FeRh/MgO by at least five times in terms of the MAE, where the MAE values are 0.24 (0.19) and 1.21 (1.27) meV/cell for the 5 *uc* (7 *uc*) FeRh/MgO and Ta/FeRh/MgO multilayers, respectively. In addition, our calculations show that the AFM order at the Ta/FeRh interface [i.e., AFM(I_Ta_)/FM(I_O_)] results in large negative MAE of −2.47 meV/5 *uc* (−2.54 meV/7 *uc*), which indicates that the magnetic state of FeRh at the interface is decisive factor in determining the sign of MAE, i.e., preferable magnetization direction.

In addition to the magnetocrystalline anisotropy, we estimate the contribution of the shape anisotropy energy (or demagnetization energy) to the measurable magnetic anisotropy of FeRh films. We calculate the shape anisotropy, ${K}_{s}$, by summing the magnetostatic dipolar interactions between the magnetic moment densities of the Fe and Rh atoms, ${K}_{s}=(\,-\,1/2){\mu }_{0}{M}_{s}^{2}$, where *μ*_0_ is the permeability constant, and *M*_*s*_ is the saturation magnetization^27^. The calculated *K*_*s*_ values are −0.07 and −0.21 meV/cell for the 5 *uc* FeRh/MgO and Ta/FeRh/MgO multilayers, respectively, the magnitude of which decreases gradually with the thickness of FeRh films owing to the localization of the ferromagnetic nature within the interface layers. In an ultrathin film, for the FeRh/MgO bilayer, the contribution of the shape anisotropy to the total MAE thus becomes significant, leading to the negligibly small MAE.

The crucial role of the interfacial atoms on the large PMA is further revealed from the atom-to-atom decomposition of the spin-orbit coupling (SOC) energy difference for in- and out-of-plane magnetization (*M*) orientations, ${\rm{\Delta }}{E}_{soc}={E}_{soc}({M}^{\parallel })-{E}_{soc}({M}^{\perp })$. Here, ${E}_{soc}=\langle \frac{{\hslash }^{2}}{2{m}^{2}{c}^{2}}\frac{1}{r}\frac{dV(r)}{dr}\hat{L}\cdot \hat{S}\rangle$, where *V*(*r*) is the spherical part of the effective potential within the PAW sphere, and $\hat{L}$ and $\hat{S}$ are orbital and spin operators, respectively. These expectation values are twice the actual value of the total energy correction to the second order in the SOC^28,29^, i.e., $MAE\approx \frac{1}{2}{\rm{\Delta }}{E}_{soc}$. The other 50% of the SOC energy translates into the crystal-field energy and the formation of the unquenched orbital moment^30^. It should be noted that the MAE is not precisely localized at the atomic site, and thus, the atom-resolved contributions do not simply add up, as previously discussed in the literature^31–33^. Nevertheless, the sum of the layer-resolved contributions calculated from the above method is in reasonable agreement (within 10%) with those obtained from total energy calculations for the whole system. The calculated MAEs for each of the FeRh and Ta(I) layers are shown in Fig. 4(b) for FeRh, FeRh/MgO, and Ta/FeRh/MgO multilayers. In all the systems, the MAE is considerable exclusively for the interface layers, whereas those of the centre layers are rather small, maintaining the high symmetry bulk-like feature. The Fe(I_V_) layer provides a dominant contribution to the negative MAE of FeRh films, whereas this contribution is smaller and positive for Rh(I), in agreement with previous full-potential calculations^21^. This negative MAE of the Fe(I_V_) layer is reversed in sign in the presence of MgO, which is due to the magnetic-phase transition and orbital hybridization between the Fe 3*d* and O 2*p* states at the interface, as mentioned previously. Thus, the small positive MAE of the FeRh/MgO bilayer is the result of the opposite contributions from the Fe(I_V_) and Fe(I_O_) layers. Similarly, the presence of the Ta capping layers results in a positive MAE for the Fe(I_Ta_) layer. Moreover, the Ta(I) layer contributes positively to the total MAE, and thus, the large PMA is preserved for Ta/FeRh/MgO.

**Figure 5:**
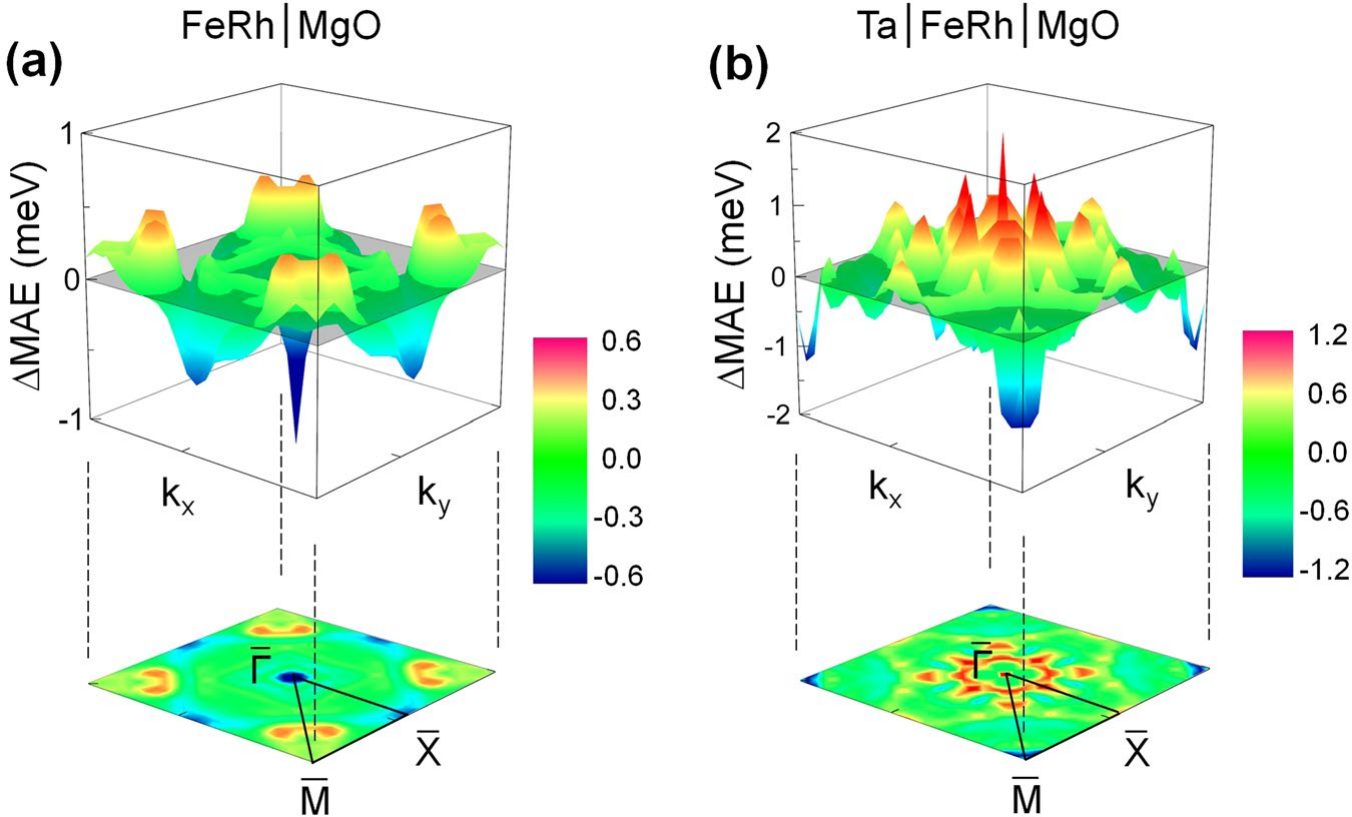
Change in MAE distribution over *k* space, ΔMAE_k_, (top) and its projection onto the two-dimensional Brillouin zone (bottom) of (**a**) FeRh in the presence of MgO and (**b**) FeRh/MgO in the presence of Ta capping. The energy scale in the bar is in units of meV.

In Fig. 5(a,b), we show the changes of the MAE distribution over *k* space, ΔMAE_k_, for FeRh in the presence of MgO, ΔMAE_k_ = MAE_k_(FeRh/MgO) - MAE_k_(FeRh), and in the presence of Ta capping, ΔMAE_k_ = MAE_k_(Ta/FeRh/MgO) - MAE_k_(FeRh/MgO), respectively. Here, MAE_k_ is calculated using the force theorem^34^; $${{\rm{MAE}}}_{{\rm{k}}}=$$$$\sum _{o}[\varepsilon {(n,k)}^{\parallel }-\varepsilon {(n,k)}^{\perp }]$$, where $$\varepsilon {(n,k)}^{\parallel }$$ and $$\varepsilon {(n,k)}^{\perp }$$ are the eigenvalues of occupied states in the Hamiltonian for in-plane and perpendicular magnetizations, respectively. By taking the sum of MAE_k_ over *k*-points, we find MAE values of −0.34, 0.32, and 1.41 meV/cell for FeRh, FeRh/MgO, and Ta/FeRh/MgO, respectively, which fairly reproduce those obtained by the total energy method [Fig. 4(a)]. For FeRh/MgO, while the MAE_k_ around the $$\bar{{\rm{\Gamma }}}$$ and $$\bar{{\rm{X}}}$$ points provide the main negative contributions to the MAE[Fe(I_V_)], the positive MAE of Fe(I_O_) is mainly predominated by the MAE_k_ distributions around the $$\bar{{\rm{M}}}$$ point. As seen in Fig. 5(b), these positive contributions of Fe(I_O_) around $$\bar{{\rm{M}}}$$ remain almost unchanged in the presence of the Ta capping layers (i.e., negligibly small ΔMAE_k_). On the other hand, the distribution of ΔMAE_k_ is rather unlocalized with a different sign over the Brillouin zone (BZ) surface for the Ta/FeRh/MgO multilayer. Notably, the aforementioned negative contributions of Fe(I_V_) around the $$\bar{{\rm{\Gamma }}}$$ and $$\bar{{\rm{X}}}$$ points, especially for $$\bar{{\rm{\Gamma }}}$$, disappear in the presence of Ta, which, in turn leads, to the large PMA of Ta/FeRh/MgO. Thus, the following discussion regarding the origin of the large PMA of Ta/FeRh/MgO will mainly focus on the SOC states around the BZ $$\bar{{\rm{\Gamma }}}$$ point.

**Figure 6:**
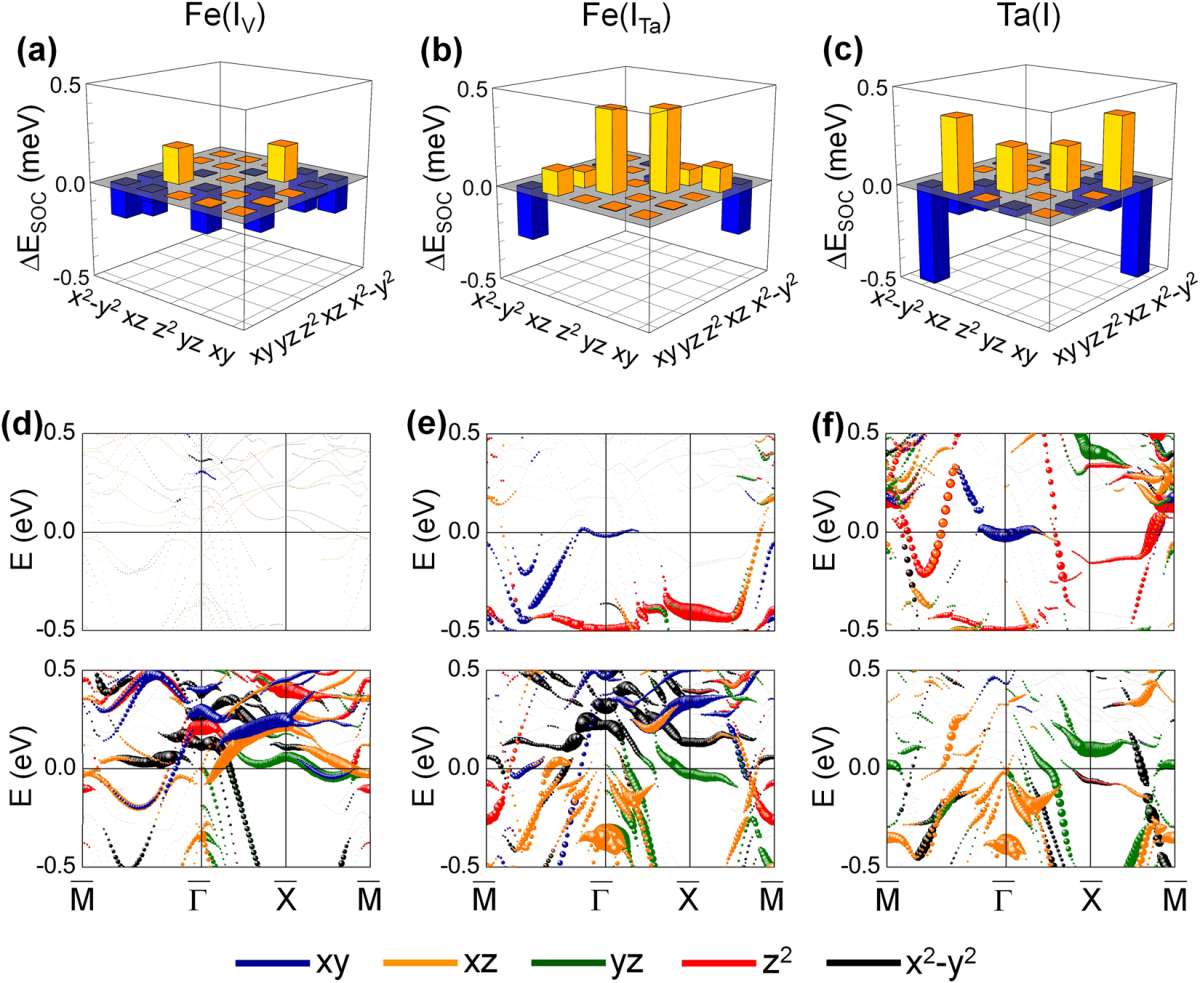
The *d*-orbital matrix decomposed Δ*E*_soc_ of (**a**) Fe(I_V_) for FeRh/MgO and (**b**) Fe(I_Ta_) and (**c**) Ta(I) for Ta/FeRh/MgO multilayers. (**d**–**f**) The corresponding *d*-orbital projected band structures in the majority-spin (top panels) and minority-spin (bottom panels) states. The *d*_xy_, *d*_xz_, *d*_yz_, *d*_z_^2^, and *d*_x_^2^ − _y_^2^ orbital states are shown in blue, orange, green, red, and black symbols, respectively. The sizes of the symbols represent the weights of the *d*-orbital states. The Fermi level is set to zero energy.

To gain more insights, we show the ${\rm{\Delta }}{E}_{soc}$ projected onto the *d*-orbital matrix elements of Fe(I_V_) for FeRh/MgO and Fe(I_Ta_) and Ta(I) for Ta/FeRh/MgO in Fig. 6(a–c). The corresponding *d*-orbital projected majority-spin (top panels) and minority-spin (bottom panels) band structures along $\overline{{\rm{M}}{\rm{\Gamma }}{\rm{X}}{\rm{\Gamma }}}$ are also shown in Fig. 6(d–f). In second-order perturbation theory, the MAE is determined by the SOC between occupied and unoccupied bands^35^; $MA{E}^{\sigma \sigma \text{'}}={\xi }^{2}{\sum }_{o,u}\frac{{|\langle {\Psi }_{o,\sigma }|{L}_{z}|{\Psi }_{u,\sigma \text{'}}\rangle |}^{2}\,-\,{|\langle {\Psi }_{o,\sigma }|{L}_{x}|{\Psi }_{u,\sigma \text{'}}\rangle |}^{2}\,}{{E}_{u,\sigma \text{'}}-{E}_{o,\sigma }}$, where ${\Psi }_{o,\sigma }$ (${\Psi }_{u,\sigma \text{'}}$) and ${E}_{o,\sigma }$ (${E}_{u,\sigma \text{'}}$) are the eigenstates and eigenvalues of occupied (unoccupied) states for each spin state, $\sigma ,\sigma \text{'}=\uparrow ,\downarrow$, respectively, and *L*_*x*(*z*)_ is the *x* (*z*) component of the orbital angular momentum operator. For $\sigma \sigma \text{'}=\uparrow \uparrow$ or $\sigma {\sigma }^{\text{'}}=\downarrow \downarrow$, the positive (negative) contribution to the MAE is determined by the SOC with the same (different by one) magnetic quantum number (*m*) through the *L*_*z*_ (*L*_*x*_) operator. For $\sigma \sigma \text{'}=\uparrow \downarrow$, the MAE has the opposite sign, so the positive (negative) contribution comes from the *L*_*x*_ (*L*_*z*_) coupling.

By analysing the *k* and *d*-orbital decomposed MAE along with the band structure, the negative MAE of the Fe(I_V_) site is predominated by the SOC term with the matrix element $\langle xz,yz\downarrow |{L}_{x}|{z}^{2}\downarrow \rangle$ at the $\bar{{\rm{\Gamma }}}$ point, which is the most dominant contribution over the other negative contributions, associated with the minority-spin ${d}_{xy}$, ${d}_{xz,yz}$, and ${d}_{{x}^{2}-{y}^{2}}$ states, and the positive contribution by $\langle xz,yz\downarrow |{L}_{z}|yz,xz\downarrow \rangle$. The other spin-channel contributions of the spin up-up ($\uparrow \uparrow$) and spin up-down ($\uparrow \downarrow$) to the MAE can be simply neglected due to the completely filled majority-spin states. This negative contribution is essentially not present for both the Fe(I_O_)^5^ and Fe(I_Ta_) sites because of the absence of the minority-spin empty ${d}_{{z}^{2}}$ state at $\bar{{\rm{\Gamma }}}$ (due to the hybridization with the O-*p* and Ti-*d* orbital states). Instead, the positive term due to $\langle {z}^{2}\uparrow |{L}_{z}|xz,yz\downarrow \rangle$ (above and below $\bar{{\rm{\Gamma }}}$) becomes more dominant in the Fe(I_Ta_) site. Moreover, the majority-spin *d*_*xy*_ band around the Fermi level can also provide an additional positive contribution by coupling with the minority-spin ${d}_{xz,yz}$ bands through $\langle xy\uparrow |{L}_{z}|xz,yz\downarrow \rangle$. One can apply these arguments to the Ta(I) case because of the feature similarities in band characters (around $\bar{{\rm{\Gamma }}}$) and SOC matrix elements. In addition, the coupling between the in-plane orbital states, ${d}_{xy}$ and ${d}_{{x}^{2}-{y}^{2}}$, with the opposite spin channel always provides a negative contribution for all interfaces, which decreases gradually from the Ta(I) to the Fe(I_V_) site, as shown in Fig. 6(a–c).

## Conclusion

Through electronic structure first-principles calculations, we have predicted that the presence of heavy transition-metal capping has a tremendous effect on the interfacial magnetism and magnetic anisotropy of Ta/FeRh/MgO multilayers. The Ta capping layers can lead to the AFM-FM transition of the Fe layers at the interface, where the magnetic phase is determined by the mutual mechanisms of the capping-induced volume/tetragonal expansion and the competition between the magnetic exchange couplings, that is, the direct and indirect interaction. Through single-particle energy spectra analyses with spin-orbit Hamiltonian matrix elements, we have further provided the physical origin of the large PMA found in Ta/FeRh/MgO, where strong hybridizations of the Fe 3*d*–O 2*p* and Ta 5*d*–Fe 3*d* orbital states at both interfaces are prominent. This system can act as a prototype for in-depth studies of the microscopic origin of the large PMA in tunnel junctions of heavy metal capped antiferromagnetic films on MgO, which would motivate further experimental investigations of the feasibility of antiferromagnetism in perpendicular memory devices.

## Methods

Density functional theory (DFT) calculations were performed using the projector augmented wave (PAW) pseudopotential method^36^, as implemented in the Vienna *ab initio* simulation package (VASP)^37,38^. The exchange and correlation interactions between electrons were described with the generalized gradient approximation (GGA) formulated by Perdew, Burke, and Ernzerhof (PBE)^39^. We used an energy cutoff of 500 eV and a 15 × 15 × 1 Brillouin zone *k*-point mesh to relax the structures until the largest force decreased to below 10^−2^ eV/Å and the change in the total energy between two ionic relaxation steps was smaller than 10^−5^ eV. The MAE was obtained based on the total energy difference when the magnetization directions were in the *xy* plane (${E}^{\parallel }$) and along the *z*-axis (${E}^{\perp }$): MAE = ${E}^{\parallel }$ − ${E}^{\perp }$. To obtain reliable MAE values, the Gaussian smearing method with a smaller smearing of 0.05 and dense *k*-points of 31 × 31 × 1 were used in the non-collinear calculations, for which the convergences of the MAE results with respect to the number of *k*-points and cutoff energy have been ensured. The SOC term was included using the second-variation method employing the scalar-relativistic eigenfunctions of the valence states^40^.
